
JOURNAL INFORMATION
==============================
NLM Title Abbreviation: Br J Cancer
Journal ID: Br J Cancer
NLM Journal ID: 0370635

British Journal of Cancer

ISSN: 0007-0920
EISSN: 1532-1827
Publisher: Nature Publishing Group

ARTICLE INFORMATION
==============================
PMCID: PMC2376293
DOI: 10.1038/sj.bjc.6600721
Article ID: 6600721
Article version: 1
Subjects: Keyword Index for Volume 87

Keyword Index

Print publication date: 2002 Dec 2
Electronic publication date: 2002 Dec 2
Volume: 87
Issue: 12
First page: 1488
Last page: 1491
Copyright: Copyright © 2002 Cancer Research UK
Copyright year: 2002
Copyright holder: Cancer Research UK
License: This article is licensed under a Creative Commons Attribution 4.0 International License, which permits use, sharing, adaptation, distribution and reproduction in any medium or format, as long as you give appropriate credit to the original author(s) and the source, provide a link to the Creative Commons license, and indicate if changes were made.The images or other third party material in this article are included in the article’s Creative Commons license, unless indicated otherwise in a credit line to the material.If material is not included in the article’s Creative Commons license and your intended use is not permitted by statutory regulation or exceeds the permitted use, you will need to obtain permission directly from the copyright holder. To view a copy of this license, visit https://creativecommons.org/licenses/by/4.0/.
License URL: https://creativecommons.org/licenses/by/4.0/

==============================

British Journal of Cancer (2002) 87, 1488–1491
© 2002 Cancer Research UK All rights reserved 0007–0920/02 $25.00
www.bjcancer.com

### Keyword Index for Volume 87

δ-aminoleuvinic acid 471
5-fluorouracil (5-FU) 591, 702, 1210, 1216
5-FU adjuvant chemotherapy 868
$^{14}$C-meta(tetrahydroxyphenyl)-chlorin 1470

acupuncture 314
acute lymphoblastic leukaemia 513
acute phase response 277
adenocarcinoma 533, 621, 751
ADEPT 600
adhesion 859, 1128
adjuvant 1365
adjuvant therapy 377
adolescent cancers 1267
aetiological heterogeneity 545
aetiology 513
aflatoxin-albumin adducts 966
age-period-cohort 982
AIB1 1411
ALA derivative 790
ALA hexyl ester 790
alcohol 1234
alcohol consumption 516, 519, 1227
alcohol dehydrogenase II gene (ADH2) 519
allelotyping 218
amifostine 385
aminolaevulinic 246
aminolevulinic acid, ALA 790, 1321
anaemia 268, 1066, 1341
anal cancer 61
anastrozole 334, 950
androgen 54, 1321
androgen-independent prostate cancer 720
angiogenesis 86, 119, 231, 344, 537, 772, 1105, 1182, 1301, 1422, 1454
angiopoietins 1182
anoikis 924
anoxia 1173
anthracycline 1210, 1365
anti-angiogenic 1166
antisense oligodeoxynucleotides 119
antisense technology 225
antivascular 465
APC$^{Min}$ mouse 567
apoptosis 98, 231, 319, 359, 366, 555, 805, 834, 909, 924, 1019, 1105, 1188
aromatase inhibitor 950
ascites 1182
Asian–American 54
aspirin 49
asymmetric dimethylarginine 673
atypia 1301
autoantibodies 339
autologous blood stem cell transplantation 1066
autologous bone marrow transplantation 1197

BAG-1 834, 1136
Bak 319
Barrett's oesophagus 533
BBR3464 825
Bcl-2 555
BCRP 665
BCS70 989
BETA transporters 471
biliary carcinoma 702
biliary tract cancer 187
biofunctional features 868
biomarker 763
bisphosphonates 366
bladder cancer 960, 1422
blood flow 1047
blood transfusion 314
blood–brain barrier 790
bone metastasis 720
brain neoplasms 621
brain oedema 621
brain stem glioma 945
brain tumour(s) 945, 1006, 1086
BRCA2 gene 888
breakage-fusion-bridge cycle 202
breast 1246
breast axillary node metastases 70
breast cancer 21, 54, 65, 231, 289, 423, 519, 691, 705, 805, 888, 909, 971, 977, 982, 1019, 1234, 1275, 1287, 1294, 1365, 1379, 1445, 1454
breast cancer cells 449
breast neoplasms 294, 974, 1079
breast tumours 635
breast-conserving therapy 615
butyrate 1314

CA125 1112
cachexia 580
Calcium channel blocker 1462
Calcium-binding protein 1281
camptothecin(s) 608, 665
cancer 1, 524, 659, 681, 796, 834, 854, 1099, 1341
cancer autoimmunity 339
cancer cells 225
cancer genes 1287, 1294
cancer incidence 1267
cancer registration data 1221
cancer registries 1267
cancer risk and epidemiology 1246
cancer services 1221
cancer survivors 1204
cancer therapy 129
cancer trends 1267
cancer vaccine 1006
carboplatin 258, 825
carboxylesterase 659
carcinoembryonic antigen (CEA) 600
carcinogenesis 400
cariporide 238
carmofur 591
carotenoids 960
case–control study 49, 516, 524, 956, 974, 1227, 1230
caspase-3 909
caveolin-1 1140
CBP 1162
CCK-2 receptors 567
*CCND1* 654
CD44 1314
CD95 1188
CDK inhibitors 129
cDNA microarray 1153
cell culture 187
cell cycle 129
cell line 187
cell proliferation 81
centrosome 202
cervical cancer 537, 989, 1145
cervix uteri 324
*CHEK2* 1445
chemoradiotherapy 258
chemoresistance 924
chemotherapy 158, 377, 481, 497, 659, 702, 840, 938, 1066, 1079, 1197, 1370
chemotherapy response 1145
chemotherapy-chemoprotection 113
childhood cancer 746, 1204, 1257
childhood leukaemia 740
children 511, 513, 945, 1197
cholangiocarcinoma 1449
choline 783
chromosomal radiosensitivity 1379
chromosome 21 423
chronic disease 268
chronic pancreatitis 551
chronobra 1246
circulating DNA 551
cisplatin 491, 825, 850, 938, 1047, 1066, 1360
cisplatin-refractory 729
classification of cancers 1267
cleft lip and palate 524
clinical benefit 497
clinical outcome 868
clinical trial(s) 511, 600, 1370
clinicopathological parameter 81
c-*myc* 654
co-expression pattern analysis 537
co-regulators 1411
codon 72
coffee 956
cohort studies 175, 960
collaborative reanalysis 1234
Colombia 324, 1417
colon 168
colon adenomas 567
colon cancer 348, 400, 868, 1128, 1182
colorectal 681
colorectal cancer 352, 393, 441, 574, 591, 630, 1136, 1216, 1314
colorectal neoplasms 585, 600, 756
combination chemotherapy 1072
communication skills 1
comparative multiplex RT–PCR 1308
complement 1119
complications 134
computer-supported content analysis 1
congenital malformation 524
conjunctival carcinoma 301
consultant communication 502
controlled release 608
cortisol secretion 1000
COX-2 1145
CPT-11 659
C-reactive protein 264
*C. trachomatis* 1417
CTL-precursors 796
Cuba 1253
cyclo-oxygenase 574
cyclo-oxygenase inhibitors 231
cyclo-oxygenase-2 91
cyclophosphamide 1072
*cytochrome p4501A1* 529
cytokine(s) 21, 591
cytotoxic 1034
cytotoxic oedema 621
cytotoxic T-lymphocyte 1006

D-dimers 1437
DaunoXome 15
delivery of healthcare 585
dendritic cells 414
dermatofibrosarcoma protuberans 1386
diet 31, 1230
dietary factors 37
differentiation 918
dimethylarginine dimethylaminohydrolase 673
distal tubule 1092
DNA mismatch repair 1027
docetaxel 21, 385, 846, 1072, 1210
dose-finding 1072

drug conjugate 608
drug development 933
drug metabolism 277
drug sensitivity 1027
drug treatment 491
DSA 918
ductal carcinoma 1105
ductal carcinoma in situ (DCIS) 615
DX-8951f 665

E-cadherin 187, 1034, 1281
eczema 989
*ELAC2* 905
electric fields 1257
electrochemotherapy 1047
electroporation 1047
embryonal carcinoma 624
empathy 1
endometrial carcinoma 377
endometrioid adenocarcinoma 81
endometrium 81, 1354
endothelial cells (ECs) 1454
endothelial precursor cells (EPCs) 1454
endothelin receptor B 1308
endothelium 344
epidemiology 49, 61, 324, 509, 519, 746, 960, 977
epirubicin (EPI) 497
epithelial cancers 1162
epoetin alfa 1341
Er$\alpha$ 449
ERK1/2 783
erythromycin breath-test (EBT) 277
erythropoietin 268
esters 246
etiology 294
Europe 291
expression cloning 405
extracellular matrix 1314
extrahepatic metastases 840

factor H 1119
familial breast cancer 502
Fas 1188
Fas ligand 359
fatigue 264
female 49
Fhit 441
FHL-1 1119
fibrillary astrocytoma 218
fibrinolysis 1437
fibronectin 106
fibrosarcoma 1047
filgrastim 1079
flourescence microscopy 1470
flow cytometry 344
fluorouracil 393, 1390
flutamide 716
focus groups 1099
follow-up 70, 151
follow-up study 75
fried foods 1230
fruit 960
fulvestrant 1354
fumarate hydratase 446
functional adrenal tumour 1000

G-CSF 846
G1-phase growth arrest 113
G2 assay 1379
G2 checkpoint 352
GalNAc-T3 751
GALT3 1006
gastric cancer 91, 171, 1153
gastrin 567, 574
gastro-oesophageal cancer 850
geldanamycin 783
gemcitabine (GEM) 161, 491, 497, 716, 1360
gene expression 91
gene expression profiles 449
gene therapy 1173
gene-environment interaction 519
genetic polymorphism(s) 529, 1057
genetic predisposition 1379
genetics 294
genomic deletions 892
geographical study 740
germ cell tumour (GCT) 624, 729, 1066
GF120918 665
glioblastoma multiforme 218
glioma 621, 918
glomerular 1092
glucose and lipid metabolism 580, 805
glutathione S-transferase MI-1 966
glutathione S-transferase TI-1 966
glycan 918
grading 1275
granulocyte 359
green tea 309
growth 1128
growth factors 726, 876

*Haemophilus influenzae* type b 511
*HPC2* 905
haematological cells 1328
haematopoietic stem cell transplantation 1079
haemoglobin 1341
head and neck squamous cell carcinoma 938
health behaviour 1204
health economy 151
heat shock 834
heat shock protein 90 (hsp90) 339
hepatic metastases 840
hepatitis B surface antigen 966
hepatitis B virus 314
hepatitis C virus 314
hepatocellular carcinoma 956, 1449
heterogeneity 815
HHV-8 301, 1253
hierarchical cluster analysis 251
high-dose therapy 281
high-risk breast cancer 281
histology 334
histone-acetylation 1042
HIV 301
HLA-DR 756
HNPCC 892
Hodgkin's disease 291, 381
Hong Kong 982
hormone responsiveness 1105
hormone-refractory prostate cancer 720
hormones 876
HPV 301, 319, 324, 989, 1417
HRE 1173
HTLV-1 1253
human breast cancer 1411
human papillomavirus 61, 75, 373, 1013
hyperemesis gravidarum 974
hypermethylation 187
hypertension in pregnancy 971
hypoxia 1173

ifosfamide 846
IMiDs 1166
immune 21
immunohistochemical stainings 70
immunohistochemistry 86, 441, 756, 1136, 1140, 1281
immunoliposomes 106
immunomodulatory 1166
immunophenotype 513
immunotherapy 796, 840, 1006, 1034
incidence 982
induced abortion 977
induction 1390
inequalities 1221
infection(s) 509, 513, 746
inflammation 1422
insulin 726
integrin $\alpha v\beta 6$ 348
integrins 859
interferon 181
interleukin-2 194
Interleukin-6 1437
intracellular pH 238
intratumoural heterogeneity 400
intratumoural injection 938
invasion 859
*in vitro* bioscreen 98
ionising radiation 352
irinotecan 144, 258, 729, 850, 1216
irinotecan and 5-FU combination therapy 1216
irradiation 1086
isoflavones 31
isolated limb perfusion 1462

JACC study 309
JNK 1188

K-*ras* mutants 1000
K-ras 898
kallikreins 6, 763, 1287, 1294
Kaposi's sarcoma 1253
keratocanthoma 1301
Ki-67 624, 751
kinase 1128
*KLK14* 1287
*KLK15* 1294
*KRAS2* mutations 551
KSHV 301

La Hague 740
LAP 859
laryngeal cancer 516, 1227, 1230
larynx 711
leiomyomas 446
leiomyosarcomas 446
length of gestation 289
leukaemia 511, 994, 1257, 1328
limited sampling 144
lipid mobilising factor 580
liposomal daunorubicin 15
lobular carcinoma 1105
locally advanced breast cancer 1404
loss of heterozygosity 218, 432, 1013, 1449
LSIL 1417
lung cancer 43, 212, 481, 694, 751, 1013
lung metastasis 359
lung neoplasm(s) 49, 585, 1112
lymph node metastasis 171
lymphadenectomy 377
lymphocytes 194
lymphoscintigraphy 705

MAG-CPT 608
malignancy 846
malignant melanoma 151
malignant mesothelioma 491
mammographic density 876
mammography screening 65
MAP kinase 348
marimastat 161
maternal hormones 545
matrix metalloproteinase 348
megatherapy 1197
MEK 1000
melanocortin 1 receptor 414
melanoma 181, 366, 1431
melanoma antigen 414
membrane transport 471
menstrual cycle 373
meta-analysis 694, 1086
metabolism 681
metallothionein 533, 1019
metastasis 231, 1197, 1308, 1314, 1328
metastatic breast cancer 15, 1072, 1210
methionine-dependence 225
methylenetetrahydrofolate reductase 225
MGMT 168
microdissection 432
micrographic controlled excision 1386

© 2002 Cancer Research UK

micrographic surgery 1386
micronucleus (MN) assay 1379
microsatellite 1449
microsatellite instability 400, 756
microsatellite marker 432
microvessel count 694
microvessel density 344, 694
migration 54, 859
mismatch repair 400, 892
mitomycin 850
Mlh1 441
MMP-9 859
molecular diagnosis 1449
monitoring 1221
monoclonal antibody 600, 1119
monocytic leukaemia 509
morphometry 1275
mortality 31, 37, 291
Msh2 441
mTHPC fluorescence spectroscopy 1470
Mts1 1281
Muir–Torre syndrome 892
multidrug resistance 665
mutation 187, 208, 446, 888, 1445
mutation analysis 635
mutations 1162

N-MYC 779
N0 neck 711
$Na^+$-dependent $Cl^-/HCO_3^-$ exchanger 238
$Na^+/H^+$ antiport 238
natural history 75
natural immunity 1119
natural killer cell 194
NCDS 989
neoadjuvant 334
neoadjuvant therapy 950
neoplasm staging 1112
neoplasms 268
nephrotoxicity 1092
neuroblastoma 1197
neutrophil 1370
NICE 481
nifedipine 1462
nitric oxide 673
nitric oxide synthase 673
node-negative breast cancer 772
non-axillary sentinel node 691
non-platinum chemotherapy 1341
non-seminoma 545
non-small cell lung cancer (NSCLC) 158, 251, 258, 264, 385, 481, 825, 1360
normal adrenocortical cells 1000
NTRK1 645
nutritional status 1370

$O^6$-alkylguanine DNA-alkyl-transferase 168
occult metastases 711
ocular melanoma 840
oesophageal cancer 529
oesophageal squamous cell carcinoma 883
oestrogen receptor 423
oestrogen receptor antagonist 1354
oestrogen(s) 54, 950
oncogenes 654
OR isoforms 1411
oral chemotherapy 933
oral squamous carcinoma 1396
oral supplementation 113
outcome 779
ovarian cancer 86, 339, 763, 815, 1119
ovarian cancer xenografts 665
oxaliplatin 393, 702, 825
oxygen pressure 1047

$p21^{waf1}$ 1042
p27 81
p300 1162
*p53* 1013
p53 441, 1019, 1027
paclitaxel 21, 681, 815
palliative care 733
palliative chemotherapy 729
pamidronate 366
pancreatic adenocarcinoma 551
pancreatic cancer 161, 555, 716, 850, 898, 1034, 1140
participation 562
pathogenesis 294
pathology 334
patient selection 615
patients' preferences 854
PCAF 1162
PDT 246
peptide vaccine 796
performance status 264
perinatal exposures 545
peripheral blood lymphocytes 1379
peripheral blood stem cell 1197
peritoneal dissemination 1153
pharmacodynamics 144, 783
pharmacokinetics 144, 608, 1072, 1470
pharmacology 1354
pharynx 711
phosphocholine 783
phospholipids 1370
photodynamic 246
photodynamic therapy, PDT 471, 790, 1321
photosensitiser 1470
physiology 1246
platin 1390
platinum 815
pleomorphic adenoma 202
PMS2 1027
poisson distribution 982
poisson regression 740
polymer 608
polymorphisms 212, 905, 1013
postmenopausal 334, 1354
postmenopausal breast cancer 950
PPAR$\gamma$ 1396
pre-eclampsia 971
pre-vaccination 796
predictive factors 1287
predictive markers 630, 1294
predictors 502
pregnancy 75, 289
prenatal origin 994
preneoplasia 432
prevalence 75, 373, 1253
proctitis 134
prodrug 659
prognosis 8, 151, 654, 751, 763, 883, 1112, 1136, 1140, 1145, 1275, 1422
prognostic factors 8, 194, 1066, 1086, 1281, 1287, 1294, 1404
prognostic index 8
prognostic significance 70
prognostic value 772
programme evaluation 733
proliferation 1105
prostaglandin $J_2$ 1396
prostate 1188
prostate cancer (PrCa) 905
prostate cancer progression 726
protein kinase B/Akt 924
protophorphyrin-IX 246
proximal tubule 1092
*PTEN/MMAC1* 1431
*P urinaria* 98

qualify of life 264, 491, 720, 1341

radiotherapy 134, 385
RAF 1000
raltitrexed 591
randomisation 854
randomised clinical trial 615, 938
randomised trial 562
real-time quantitative polymerase chain reaction (RQ–PCR) 779
real-time reverse transcriptase polymerase chain reaction 883
RECIST 158
record linkage 524
recurrence 377, 938, 1386
reduced risk 28
regression analysis 1221
relapse 729
renal cell carcinoma 119, 194
reproductive outcome 381
reproductive variables 1246
resistance 681
resistance factors 251
response 158
retinoblastoma 779
retinoids 555, 1321
retrotransposon 1173
Rho GTPases 635
Rho proteins 366
right-sided colon 400
risk factors 281, 533, 956, 1227, 1230, 1253, 1417
ROA 1411
RT–PCR 181
rural population mixing 740

S100A4 1281
S3705 238
salivary gland cancer 1042
SC142 antibody 405
SC142 scFv 405
SC142-reactive antigen 405
scFv antibody 106
screening programmes 65
seasonality 509
second-line chemotherapy 1210
SelCIDs 1166
semen cryopreservation 381
seminoma 545, 624
sentinal node 711, 705
serine proteases 1287, 1294
SERMs 449
serological analysis of recombinant cDNA expression libraries (SEREX) 339
serotonin 465
Shc 645
signal transduction 645
signalling 630
SMAD 630
smoking 516, 1234
smoking cessation 1227
SN-38 144, 659
social problems 1099
socioeconomic factors rural population urban population 585
solid tumours 746
somatostatin 86
somatostatin receptors 86
South Korea 314
soybeans 31
SP-B 212
Sp1 1042
space-time clustering 513
squamous 319
squamous cell carcinoma 202, 212, 529, 711, 859, 1470
SRA 1411
Src 1128
ST7 gene 208
stage migration 171
staging 705
Stat3 1396
staurosporine 909
stealth liposomal doxorubicin 385
steroid hormones 1294
steroid receptors 8
stomach cancer 31, 37, 309, 888
suppression subtraction hybridisation 423

© 2002 Cancer Research UK

surfactant 212
survival 264, 562, 756, 763, 1086, 1365
*survivin* 91, 883
susceptibility 905
synovial sarcoma 28
systematic review 134
systemic inflammation 591

T cells 1034
Tamoxifen 1411
TaqMan PCR 1328
TEL-AML 1 fusion gene 994
telomerase 98
telomere 202
testicular cancer 545
testis 624
TGF$\beta$ 630
TGF-$\beta$1 859
thalidomide analogues 1166
thiazolidinediones 1396
time-dependent 8
tissue 681
tissue polypeptide antigen 1112
TNM-UICC classification 171
tobacco 1234
topoisomerase 624
total body irradiation 1197
toxicity 846
training 1
transcription 834, 1042
transforming activity 645
transforming growth factor $\beta$2 113
transitional cell carcinoma 654
treatment 691, 1086
treatment outcome 733
trends 43, 291
triplet 1360
TRK-T3 645
trophic properties 574
trophosphamide 945
tubulin 98
tumour 950
tumour angiogenesis 1057
tumour antigens 339, 1006
tumour burden 994
tumour cell invasion 537
tumour immunology 414
tumour markers 414, 1112, 1287, 1294, 1404
tumour metabolism 805
tumour necrosis factor 465
tumour oxygenation 1462
tumour perfusion 1462
tumour progression 635
tumour response 497
tumour suppressor gene 208, 432, 1431
tumour targeting 106
tumour vascular resistance 1462
tumour virology 175
twins 294
tyrosinase mRNA 181

UCN-01 352
Uganda 301
UI small nuclear RNA 898
UVB 319
uveal melanoma 1308

vaccinations 511
vascular endothelial growth factor 119, 673, 772
vascular permeability 1182
vascularisation 1301
vasculogenesis 1454
vasogenic oedema 621
vegetables 960
VEGF 1301
VEGF-A 1437
vesnarinone 1042
vinorelbine 1210, 1360
viral persistence 175
vitamin D 1321
vitamins 960
VL30 1173
VPI6 945

warts 989
weight loss 264, 1370
WHO 158
Will Rogers phenomenon 171
Wilms' tumour 1092

X-chromosomal inactivation 28
xenografts 1328

young adult cancers 1267
young adults 1204

ZD0473 825

© 2002 Cancer Research UK
